# Towards 90-70-90 targets: Individual and community level factors associated with cervical cancer screening among women of reproductive age in Tanzania: A multi-level analysis based on 2022 Tanzania demographic and health survey

**DOI:** 10.1371/journal.pone.0315438

**Published:** 2024-12-18

**Authors:** Yordanos Sisay Asgedom, Aklilu Habte Hailegebireal, Beshada Zerfu Woldegeorgis, Mengistu Meskele Koyira, Beminate Lemma Seifu, Bezawit Melak Fente, Amanuel Yosef Gebrekidan, Habtamu Azene Tekle, Angwach Abrham Asnake, Gizachew Ambaw Kassie

**Affiliations:** 1 Department of Epidemiology, College of Health Sciences and Medicine, Wolaita Sodo University, Sodo, Ethiopia; 2 School of Public Health, College of Medicine and Health Sciences, Wachemo University, Hosanna, Ethiopia; 3 Faculty of Health and Environmental Sciences, Auckland University of Technology, Auckland, New Zealand; 4 School of Medicine, College of Health Sciences and Medicine, Wolaita Sodo University, Sodo, Ethiopia; 5 School of Public Health, College of Health Sciences and Medicine, Wolaita Sodo University, Sodo, Ethiopia; 6 Department of Public Health, College of Medicine and Health Science, Samara University, Samara, Afar, Ethiopia; 7 Department of General Midwifery, School of Midwifery, College of Medicine & Health Sciences, University of Gondar, Gondar, Ethiopia; University of Nairobi, KENYA

## Abstract

**Introduction:**

Cervical cancer is a major public health problem worldwide, and is mainly caused by human papillomaviruses. More than 90% of cervical cancer cases can be prevented by using a human papilloma vaccine and screening. Despite the ongoing global cervical cancer screening target, uptake remains unacceptably low in sub-Saharan Africa such as Tanzania. Although cervical cancer is the leading cause of mortality in Tanzania, evidence on the individual- and community-level factors associated with cervical cancer screening among women of reproductive age is scarce. Therefore, this study aimed to determine the individual- and community-level factors associated with cervical cancer screening among women of reproductive age in Tanzania.

**Methods:**

This study used data from the 2022 Tanzania Demographic and Health Survey (TDHS). A weighted sample of 15,140 women of reproductive age was included in this study. Given the effect of clustering and binary nature of the outcome variable, we used a multilevel binary logistic regression model. The adjusted odds ratio (AOR) with 95% Confidence Interval (CI) was statistically significant. Moreover, the model with the lowest deviance best suited the data.

**Results:**

The overall uptake of cervical cancer screening among Tanzanian women was 7.28% (95% confidence interval [CI]: 6.87%, 7.70%). Women’s age (25–34, 35–49), women with primary, secondary, and higher educational levels, being employed, a high household wealth index, visiting health facilities in the last 12 months, owning mobile phones, urban residence, and southern highlands, Southern, and Zanzibar administrative zones, were significantly associated with cervical cancer screening.

**Conclusion:**

Cervical cancer screening among women in Tanzania was low. Low uptake underscores the need for increased focus on addressing the coverage of the 2030 Sustainable Development Goals (SDGs). The study would help policymakers create programs that consider education, employment, visiting health facilities, mobile phones, wealth, residence, and administrative zones, which would make women undergo cervical cancer screening. Pointing to women living with low cervical cancer screening could help increase their uptake and achieve the targets of the national and World Health Organization.

## Introduction

Cervical cancer is the fourth most common cancer in women, and globally, approximately 7.5% of cancer-related deaths among women are attributed to it [1–3]. Cervical cancer is caused by persistent human papillomavirus (HPV). Human papillomavirus (HPV) is one of the most common sexually transmitted diseases worldwide. HPV has more than 130 low- and high-risk serotypes, and high-risk serotypes (HPV 16 and 18) can cause cancers such as cervical cancer, although the low risk causes benign warts [4]. There is a risk for all women that HPV infection may become chronic and that precancerous lesions may progress to invasive cervical cancer [1].

In 2020, an estimated 604,000 women were diagnosed with cervical cancer, and approximately 342,000 deaths from the disease occurred worldwide, with low- and middle-income countries accounting for more than 90% of the deaths [5, 6]. In sub-Saharan Africa (SSA), cervical cancer is the second leading cause of cancer in women, accounting for 22.5% of all cancer cases. Moreover, approximately 117,316 females are diagnosed annually with the disease [7]. In SSA, the overall prevalence of cervical cancer screening is low [8]. Tanzania is one of the five countries with the highest cervical cancer rates in Africa [9]. The prevalence of cervical cancer in Tanzania is a significant health issue, with high global rates and the third highest mortality rate among countries eligible for support from the GAVI Alliance [10]. Tanzania has a prevalence of 89,693 (0.14%) cancer cases by 2022, with an annual incidence of 44,931(0.07%) and 29,743 (0.04%) cancer-related deaths [11]. However, the prevalence of cervical cancer in Tanzania is significantly higher, with 10,868 cases (24.2%), making it the most common cancer [11]. Moreover, the prevalence of cervical cancer in Malawi was lower than that in Tanzania (4701 (23.7%]). Cervical cancer predominantly affects young, tobacco smokers, those with a history of multiple sexual partners, those who have sexually transmitted diseases, immunocompromised, and uneducated females living in less developed countries where access to information about the disease, screening, and treatment is inadequate [6].

HPV vaccination and regular screening are recommended to reduce the burden of cervical cancer in low-income and middle-income countries [12]. However, ample gaps exist due to patient-level factors, health systems, and providers [13]. Owing to the higher risk of HPV positivity at the age of 30, the World Health Organization (WHO) recommends initiating cervical cancer screening every 5–10 years. Moreover, it encourages a minimum of two lifetime screens with a high-performance HPV test by the age of 35, and again by the age of 45 [1]. Lugol’s iodine (VILI), acetic acid (VIA), and Papanicolaou tests (Pap smear) are the simplest visual tests for cervical cancer screening [14]. They have all been proven to be effective for cervical cancer screening, although there are differences in the screening methods [14, 15].

In Africa, cervical cancer screening is available only in selected public health facilities, which presents the challenge of accessing women in rural areas [9]. Although cervical cancer screening in Tanzania is free of charge in government and church-based hospitals using VIA [16], the uptake of the screening program remains low, particularly in rural populations and those with low socioeconomic status. The low uptake of cervical cancer screening ranges from 7.9% to 8.2% in Tanzania [17, 18].

The WHO has established–90-70-90 targets for 78 low-income and lower-middle-income countries. These goals include (1) ensuring that 90% of girls receive proper HPV vaccination, (2) screening 70% of women for cervical cancer, and (3) providing treatment to 90% of women diagnosed with cervical cancer by the end of 2030. All countries have committed to eliminating cervical cancer as a public health problem by 2030, with the aim of screening 70% of women with a high-quality test by the ages of 35 and 45 [1]. Since 2002, the Tanzanian Ministry of Health and Social Welfare [19] has been working in collaboration with various partners, including the WHO, the International Agency for Research on Cancer (IARC), the international non-profit Jhpiego, and numerous local and international non-governmental organizations, to increase efforts aimed at preventing cervical cancer among Tanzanian women [3]. Cervical cancer screening is a critical factor in addressing the burden of the disease. Therefore, evidence on the determinants of cervical cancer screening is a burning issue. However, there is a paucity of data on individual- and community-level factors in cervical cancer screening in Tanzania. Prior studies have revealed that poor knowledge of cervical cancer screening, unfavorable religious attitudes, and the unavailability of screening services at women’s usual healthcare points significantly impact the uptake of cervical cancer screening [20, 21]. However, existing studies have limitations such as small sample sizes, data derived from facility-based surveys lacking national scope/relevance, and failure to consider community-level factors. This study aimed to examine the relationship between individual, household, and community factors and cervical cancer screening uptake among women of reproductive age in Tanzania, using multi-level regression analysis. These findings could aid in designing evidence-based interventions and measures aimed at improving the availability and accessibility of cervical cancer screening in low-resource settings (Tanzania). Policymakers, healthcare providers, and researchers in Tanzania can use these findings to make evidence-based decisions and implement interventions that help improve the screening uptake level.

## Materials and methods

### Data source and sampling procedure

This study was based on the 2022 Tanzania Demographic and Health Survey. The TDHS is conducted every five years to update the health and health-related indicators. The data were derived from the Demographic and Health Survey (DHS) program, and detailed information about the surveys can be found in the DHS reports for each country. A two-stage stratified sampling technique was used to select the study participants. In the first stage, Enumeration Areas (EAs) were randomly selected, and households were selected in the second stage. There are different datasets in the DHS; for this study, we used the Individual Record (IR) file. The dependent and independent variables were extracted from the IR dataset based on literature. A total weighted sample of 15,140 women aged 15–49 years was included in the final analysis.

### Study variables and measurements

#### Dependent variable

The outcome variable of interest was screening for cervical cancer. Cervical cancer screening was measured in terms of whether respondents underwent any cervical examination ever; respondents were specifically asked “Have you ever been tested or examined for cervical cancer by healthcare provider?” (No, Yes). It was categorized as ‘‘YES” if women tested or examined for cervical cancer and ‘‘No” otherwise.

#### Independent variables

Because of the hierarchical nature of the DHS data, the independent variables considered in this study were obtained from two sources (individual- and community-level variables). Women’s age, education level, employment status, number of children, modern contraceptive use, mobile phone use, Internet use, media exposure, health insurance coverage, household wealth status, visiting health facilities in the past 12 months, having permission to access health care, ease of money to seek medical care, and distance to health facilities were level one variables that were unique for each woman. Residence, administrative zones, and autonomy in decision-making were level-two variables shared by all women residing in the same community (cluster).

### Data management and analysis

The data were weighted using sampling weight, primary sampling unit, and strata before any statistical analysis to restore the representativeness of the survey and consider the sampling design when calculating standard errors to obtain reliable statistical estimates. STATA version 17 statistical software (StataCorp, USA) was used for the data management and analysis. The outcome variables in this study were dichotomous. Therefore, a multilevel binary logistic regression model was used to analyze the predictors of cervical cancer screening.

In addition, DHS data are hierarchical in nature. Therefore, women are nested within a cluster, and we assume that the study subjects in the same cluster may share similar characteristics with those in another cluster. This violates the independence observations and equal variance assumptions between the clusters in the binary logistic regression model. This implies the need to consider heterogeneity between clusters using an advanced model. Therefore, a multilevel cumulative logit model was used.

### Model building and selection

Four models were constructed for multilevel binary logistic regression analysis. The first was a null model without explanatory variables to determine the extent of cluster variation in cervical cancer patients. The second model was adjusted for individual-level variables, the third model was adjusted for community-level variables, and the fourth model was simultaneously fitted to both individual- and community-level variables. Model comparison was made based on deviance (-2Log-Likelihood Ratio (LLR)) because the models were nested models, and the model with the lowest deviance was the best-fitted model for the data.

Variables with a p-value ≤ 0.2 in the bi-variable multilevel binary logistic regression model were considered for the multivariable analysis. In the multivariable multilevel binary logistic regression model, the Adjusted Odds Ratio (AOR) with a 95% Confidence Interval (CI) was reported to indicate the strength of the association, and the statistical significance for the final model was set at p<0.05.

### Ethical consideration

As the study was a secondary data analysis of publicly accessible survey data from the MEASURE DHS program, ethical approval or participant consent was not required. We have granted permission from http://www.dhsprogram.com to download and use the data for this study. In the datasets, there were no names of persons or household addresses recorded in the dataset.

## Results

### Study participant’s descriptive characteristics

A total of 15,140 women of reproductive age were included in this study (Table 1). Of these, 5742 (37.93%) were aged 15–24 years and 8075 (53.34%) were women who attained primary education. Approximately 9736 (64.31%) patients were included in the study. Regarding household wealth status, around 3940(26.03%) were the richest, and 14,257(94.17%) had no health insurance. More than half (55.07%) had media exposure, 9004 (59.47%) owned mobile phones, and the majority (85.66%) had never used the Internet. Nearly half of the women (53.01%) visited the health facility in the 12 months and received permission to seek medical care. The ease of money to seek medical care and distance to health facilities were not significant problems for 14061(92.87), 9655(63.77), and 10780 (71.20%) women in Tanzania, respectively.

**Table 1:** Individual and community level characteristics of the study participants in Tanzania.

Variable	Weighted frequency	Percentage
Proportion of cervical cancer screening 95%CI (7.28% (95%CI = 6.87,7.70)		
**Individual level characteristics of the study participants in Tanzania**		
**Women’s age in years**		
15–24	5743	37.93
25–34	4590	30.32
35–49	4808	31.75
**Women educational status**		
No education	2424	16.00
Primary	8076	53.34
Secondary	4427	29.24
Higher	213	1.41
**Women occupational status**		
Unemployed	5404	35.69
Employed	9736	64.31
**Number of children ever born**		
≤ 3	10,394	68.65
≥ 4	4746	31.35
**Modern Contraceptive**		
Yes	3792	25.05
No	11,348	74.95
**Household wealth status**		
Poorest	2442	16.13
Poorer	2562	16.93
Middle	2860	18.89
Richer	3336	22.03
Richest	3940	26.03
**Media exposure (N = 14,685)**		
No media exposure	6598	44.93
Has media exposure	8086	55.07
**Covered by health insurance**		
Yes	882	5.83
No	14,258	94.17
**Visited health facility last 12 months**		
Yes	8026	53.01
No	7114	46.99
**Owns a mobile telephone**		
Yes	9004	59.47
No	6136	40.53
**Internet use**		
Never	12970	85.66
Yes	2170	14.34
**Having permission to access medical care**		
Big problem	1079	7.13
Not a big problem	14061	92.87
**Ease of money to seek medical care**		
Big problem	5485	36.23
Not a big problem	9655	63.77
**Distance to health facility**		
Big problem	4360	28.80
Not a big problem	10780	71.20
**Community-level characteristics of the study participants in Tanzania**		
**Residence**		
Urban	5401	35.68
Rural	9739	64.32
**Administrative Zones**		
Eastern	4353	28.75
Central	1570	10.37
Southern	803	5.31
Western	1266	8.36
Lake	4385	28.97
South western highland	1003	6.63
Southern highland	1242	8.20
Zanzibar	518	3.41
**Autonomy in decision making**		
Low	2814	18.58
Moderate	1374	9.08
High	10952	72.34

Regarding the community-level characteristics of the study participants, 9739 (64.32%) were from rural areas, 4353 (28.75%) were from the Eastern region, 4385 (.28.97%) were from Lake, and 518 (3.41%) were from Zanzibar. Autonomy in decision-making was high in 10952 (72.34%) women in the community (Table 2).

**Table 2:** Proportion of cervical cancer screening uptake among women’s of reproductive age (15–49) in Tanzania, 2024.

Variable	Cervical cancer screening			Overall
	Yes N(%)		No N(%)	
**Women’s age in years**				
15–24	124 (11.22)	5618 (40.02)		5742
25–34	347 (31.48)	4243(30.23)		4590
35–49	632 (57.30)	4176 (29.75)		4808
**Women educational status**				
No education	76(6.88)	2348(16.73)		2424
Primary	632(57.33)	7444(53.03)		8076
Secondary	358(32.48)	4068(28.98)		4426
Higher	36(3.31)	178(1.26)		213
**Women occupational status**				
Unemployed	244(22.14)	5160(36.76)		5404
Employed	858(77.86)	8878(63.24)		9736
**Number of children ever born**				
≤ 3	638(57.92%)	9755(69.49)		10393
≥ 4	464(42.08)	4283(30.51)		4747
**Modern Contraceptive**				
Yes	384(34.81)	3408(24.28)		3792
No	719(65.19)	10,629(75.72)		11348
**Household wealth status**				
Poorest	50(4.50)	2392(17.04)		2442
Poorer	101(9.14)	2462(17.54)		2563
Middle	145(13.15)	2715(19.34)		2860
Richer	302(27.41)	3033(21.61)		3335
Richest	505(45.81)	3435(24.47)		3940
**Media Exposure(N = 14,685)**				
No media exposure	333(31.01)	6235(46.03)		6599
Has media exposure	740(68.99)	7346(53.97)		8046
**Covered by health insurance**				
Yes	160(14.49)	722(5.15)		882
No	943(85.51)	13,315(94.85)		14258
**Visited health facility last 12 months**				
Yes	706(64.06)	7321(52.15)		8027
No	395(35.94)	6718(47.85)		7113
**Owns a mobile telephone**				
Yes	922(83.61)	8082(57.57)		9004
No	181(16.39)	5955(42.43)		6136
**Internet use**				
Never	781(70.77)	12,189(86.83)		12970
Yes	322(29.23)	1848(13.17)		2170
**Having permission to access medical care**				
Big problem	63(5.63)	1016(7.24)		1079
Not a big problem	1040(94.37)	13,021(92.76)		14061
**Ease of money to seek medical care**				
Big problem	358(32.43)	5127(36.53)		5485
Not a big problem	745(67.57)	8910(63.47)		9655
**Distance to health facility**				
Big problem	223(20.25)	4137(29.47)		4360
Not a big problem	879(79.25)	9901(70.53)		10780
**Residence**				
Urban	638(57.84)	4764(33.94)		5402
Rural	465(42.16)	9273(66.06)		9738
**Administrative Zones**				
Eastern	429(38.87)	3924(27.96)		4353
Central	82(7.46)	1488(10.60)		1570
Southern	21(1.87)	783(5.58)		804
Western	53(4.84)	1213(8.64)		1266
Lake	266(24.12)	4120(29.35)		4386
South western highland	72(6.52)	931(6.64)		1003
Southern highland	152(13.78)	1090(7.77)		1242
Zanzibar	28(2.54)	488(3.47)		516
**Autonomy in decision making**				
Low	133(12.05)	2681(19.10)		2814
Moderate	122(11.06)	1252(8.92)		1374
High	847(76.87)	10,105(71.98)		10952

### Uptake of cervical cancer screening

The overall uptake of cervical cancer screening was 7.28% [95% CI: 6.87%, 7.70%]. The proportion of cervical cancer screening among women with health insurance was 18.10% and the uptake of cervical cancer screening among women who attained higher education was 17.10%. Furthermore, 14.85 proportion of women used the Internet for cervical cancer screening (Table 2).

### Random effect analysis

#### Null model

This model is an intercept-only model without predictors. We examined whether the multilevel binary logistic regression model was more significant than the single-level binary logistic regression model by using the Likelihood Ratio (LR). The LR test results were statistically significant (p<0.05), indicating that the multilevel binary logistic regression model best fitted the single-level binary logistic regression analysis. Therefore, the LR-test test suggests using a multilevel binary logistic regression model. Four random-effects models were fitted, and the final model was chosen because it had the lowest deviance value (Table 3).

**Table 3:** Multilevel analysis of factors associated with cervical cancer screening among reproductive age women in Tanzania.

Variables	Null model	Model I (with level one variables)	Model II (with level II variables)	Model III (with level 1 and level 2 variables)
**Women’s age**				
15–24		1		1
25–34		3.12(2.46,3.96)		3.14 (2.47,4.00) **
35–49		6.82(5.32,8.81)		6.99 (5.43, 9.01)**
**Women’s educational status**				
No formal education		1		1
Primary education		1.86 (1.42, 2.44)		1.86 (1.38, 2.37)**
Secondary education		1.81 (1.33, 2.46)		1.95(1.43, 2.67)**
Higher education		1.65(0.98,2.77)		1.68 (1.00,2.83)**
**Women’s employment status**				
Unemployed		1		1
Employed		1.24 (1.04, 1.46)		1.19 (1.01, 1.42)*
**Covered by health insurance**				
Yes		1.78(1.41,2.23)		1.70 (1.35,2.14)
No		1		1
**Household Wealth status**				
Poorest		1		1
Poorer		1.64(1.13,2.37)		1.52(1.05,2.20)*
Middle		1.77(1.23,2.65)		1.57(1.09,2.27)*
Richer		3.01(2.07,4.36)		2.54(1.73,3.74)**
Richest		4.06(2.71,6.08)		3.18(2.06,4.90)**
**Media Exposure**				
Yes		0.86(1.07,1.43)		0.88(0.73, 1.07)
No		1		^1^
**Visited health facility in the last 12 months**				
Yes		1.24 (1.07, 1.43)		1.25 (1.08,1.45)*
No		1		1
**Owns mobile phone**				
Yes		1.32(1.09, 1.61)		1.32(1.08,1.60)*
No		1		1
**Internet Use**				
Yes		1.33 (1.09, 1.63)		1.08(0.82, 1.19)
No		1		1
**Having permission to access medical care**				
Big problem		1		1
Not a big problem		1.12(0.79,1.58)		1.09(0.76,1.55)
**Ease of money ti seek medical care**				
Big problem		1		1
Not a big problem		0.97(0.82,1.15)		0.98(0.83,1.16)
**Distance to health facility**				
Big problem		1		1
Not a big problem		0.96(0.79,1.17)		0.92(0.75, 1.12)
**Place of residence**				
Urban			2.36(1.97,2.83)***	1.38(1.12,1.70)***
Rural			1	1
**Region**				
Eastern			1	1
Central			0.70 (0.49, 1.00)	0.80 (0.57, 1.14)
Southern			0.33 (0.19, 0.55)	0.40 (0.24, 0.67)**
Western			0.55 (0.37, 0.84)	0.90 (0.60, 1.35)
Lake			0.66 (0.50, 0.87)	0.85(0.65, 1.12)
South Western highland			0.81 (0.58, 1.13)	1.08 (0.77, 1.50)
Southern highland			1.73 (1.30, 2.29)	1.71 (1.30, 2.25)**
Zanzibar			0.58 (0.43, 0.78)	0.56 (0.41, 0.76)**
**Autonomy to make decision**				
Low			1	1
Moderate			1.54(1.18,2.01)	1.23(0.93,1.62)
High			1.13 (0.92, 1.39)	1.09 (0.88, 1.36)
**Random effect analysis results**				
**Measures of variation**				
Variance	0.83	0.4439	0.4415	0.3212
ICC	0.2022	0.1188	0.1183	0.0889
AIC	7470.2	6477.3	7310.9	6418.7
BIC	7485.4	6644.4	7402.4	6661.6
PCV	Reference	29.5%	46.8%	61.3%
MOR	2.39(2.13,2.65)			
**Model fitness (Goodness of fit)**				
LLR	-3733.1	-3216.6	-3643.4	-3177.3
Deviance (-2LLR)	7466.2	6433.2	7286.8	6354.6
LR-test	LR test vs. logit model: chibar2(01) = 48.68 Prob > = chibar2 <0.001			

P-value <0.001

** P-value < 0.05 AIC: Akaike Information Criteria, AOR: Adjusted Odds Ratio, BIC: Bayesian Information Criteria, LLR: Log-Likelihood Ratio, LR: Likelihood Ratio

Table 2 shows that the Intraclass Correlation (ICC) in the null model was 20.2%, indicating that 20.2% of the overall variability in cervical cancer screening was related to the variation between clusters. Moreover, the Median Odds Ratio (MOR) for cervical cancer screening was 2.39, indicating variability between clusters. If we randomly selected individuals from two different clusters, those in the cluster with higher cervical cancer screening had 2.39 times of odds of cervical cancer screening than those in the cluster with lower uptake of cervical cancer screening. The best-fit model was chosen based on the lowest deviance value (6354.6) (Table 3).

### Factors associated with cervical cancer screening among reproductive age women in Tanzania

Bivariable analysis was performed to identify factors associated with cervical cancer screening. Accordingly, women’s age, women’s educational status, women’s employment status, total number of children, use of modern contraceptives, household wealth status, coverage by health insurance, media exposure, visited health facilities in the last 12 months, owned mobile phones, Internet use, permission to access medical care, ease of money seeking medical care, distance to health facility, residence, region, and autonomy in decision-making were included in the multivariate analysis (p<0.2). In the multilevel multivariable mixed-effect binary logistic regression model, women’s age, educational status, employment status, household wealth status, coverage by health insurance, health facilities visited in the last 12 months, owned mobile phones, internet use, residence, and region were found to be statistically significant determinants of cervical cancer screening among Tanzanian women of reproductive age. The odds of having higher level of cervical cancer screening among women who were in the age of 25–34 and 35–49 were 3.14 times [AOR = 3.14, 95% CI: 2.47, 4.00] and 6.99 times [AOR = 6.99, 95% CI: 5.43, 9.01] higher odds compared to women’s aged 15–24. A woman’s whose educational status of primary, secondary and higher had 1.86 times [AOR = 1.86, 95% CI: 1.38, 2.37], 1.95 times [AOR = 1.95, 95% CI: 1.43, 2.67] and 1.68 [AOR = 1.68, 95% CI: 1.00, 2.83] higher odds of cervical cancer screening than women with no formal education. The odds of screening for cervical cancer among employed women were 1.19 times [AOR = 1.19, 95% CI: 1.01, 1.42] higher than their counterparts. The odds of being screened for cervical cancer among women with health insurance covered was 1.70 times [AOR = 1.70, 95% CI: 1.35, 2.14] higher than their counterparts. Women’s from poorer, middle, richer and richest household wealth status were 1.52 times [AOR = 1.52, 95% CI: 1.05, 2.20], 1.57 times [AOR = 1.57, 95% CI: 1.09, 2.27], 2.54 times [AOR = 2.54, 95% CI: 1.73, 3.74] and 3.18 times [AOR = 3.18, 95% CI: 2.06, 4.90] higher odds cervical cancer screening compared to women’s with household wealth status of poorest, respectively. Women’s who visited health facility in the last 12 months were 1.25 times [AOR = 1.25, 95% CI: 1.08, 1.45] higher odds of cervical screening uptake compared to their counterparts. The odds of screening for cervical cancer among women who owned mobile phone was 1.32 times [AOR = 1.32, 95% CI: 1.08, 1.60] compared to women with no mobile phones. The higher odds of being screened for cervical cancer among internet users were 1.08 times [AOR = 1.08, 95% CI: 0.82, 1.19] compared to non-users. Women from urban residence 1.38 times [AOR = 1.38, 95% CI: 1.12, 1.70] higher odds of cervical cancer screening compared to women from rural residents.

The odds of being screened for cervical cancer in the southern highland region was 1.7 times [AOR = 1.71, 95% CI: 1.30, 2.25] compared to women in the eastern region; however, the odds of screening among women for cervical cancer from Southern and Zanzibar decreased by 60% [AOR = 0.40, 95% CI: 0.24, 0.67] and 44% [AOR = 0.56, 95% CI: 0.41, 0.76] compared to the eastern region (Table 3).

## Discussion

Although cervical cancer is highly prevalent in Tanzania, it has not received the necessary attention. The national health policy lacks a dedicated screening strategy for cervical cancer and instead focuses primarily on infectious diseases. Even though the Tanzanian Ministry of Health and Social Welfare introduced visual inspection with acetic acid (VIA) for cervical cancer screening along with cryotherapy at more than 300 locations across the country [22], the uptake of cervical cancer screening among women of reproductive age was 7.28% [95% CI: 6.87%, 7.70%], which is far from the WHO 2030 target. Moreover, rural resident women in Tanzania have a lower uptake of cervical cancer screening than urban resident women. A possible reason could be that rural regions face various obstacles that hinder access to cervical cancer screening services, as well as broader healthcare options. Factors such as low health literacy, inadequate awareness of the available screening services, and poor infrastructure contribute to these challenges. Additionally, cultural beliefs significantly influence health-seeking behaviors, affecting the likelihood of individuals seeking cervical cancer screenings [23]. This finding is in line with studies by Ethiopia [24] and Uganda [25]. Furthermore, studies have shown that a lack of awareness and understanding of cervical cancer and its screening options significantly contribute to the low rates of screening uptake [26].

The uptake of cervical cancer screening in Tanzania (7.28%) is lower than the uptake of cervical cancer screening reported from five SSA countries [27], systematic review and meta-analysis in SSA [28], Zimbabwe (13.4%) [29] and Ethiopia (13.4%) [30]. A possible explanation might be the lack of political commitment (leaders are not prioritizing healthcare programs, and funds and resources for screening programs are transferred to other programs) [26], and sociocultural influences, such as myths about cervical cancer screening, may affect their enthusiasm to undergo screening [31]. Our finding is higher than the prevalence of cancer screening in Ghana (2.4%) [32]. This may be because there is no national policy program regarding cervical cancer screening in Ghana, which could result in a low prevalence of screening in Ghana [33].

Further, in the final model, we found that women’s age, educational status, employment, covered by health insurance, household wealth status, visiting health facilities in the last 12 months, owned mobile phones, and Internet use were significantly associated with higher odds of cervical cancer screening, whereas region was associated with lower odds of cervical cancer screening.

Women aged ≥ 25 years had higher odds of cervical cancer screening than women aged less than women aged less than 25 years. This is consistent with findings reported in Ethiopia [30], Kenya [34], Zimbabwe [29]. A possible reason might be that at this age, early signs or symptoms of cervical lesions may have led them to seek cervical cancer screening before the invasive stages [35, 36]. Moreover, at this age, most women give birth and receive health education about reproductive and sexual health issues, including cervical cancer screening.

Women’s educational status had a positive effect on the uptake of cervical cancer screening. Women who had attained primary, secondary, and higher education had higher odds of being screened for cervical cancer than women with no formal education. This is consistent with findings from Ethiopia, Nigeria, and Ghana [30, 37, 38]. A possible explanation might be that education is associated with better access and utilization of healthcare services, including cervical cancer screening [39, 40]. Moreover, having a high number of educated women may ease the dissemination of information to women with poor educational status by using different community spaces and social networks [41].

Employment was significantly associated with increased odds of cervical screening. This is in line with the findings of Ghana [42, 43]. The employment of women not only promotes financial independence, but also leads to enhanced reproductive healthcare services.

Women with a high household wealth index had increased odds of cervical screening compared with women from low households. This is consistent with the findings of Zimbabwe [29] and Kenya [34]. The uptake of cervical cancer screening services and the burden of cervical cancer are inexplicably dispersed among poor women globally. A possible explanation could be that the poorest women could struggle financially, which is a hurdle to accessing cervical cancer screening [34].

Women with a history of health facility visits in the last year had higher odds of receiving cervical cancer screening services than women with no history of health facility visits. This result is consistent with the findings of Kenya [34] and Peru [44]. A possible explanation could be that to effectively access screening services, it is essential for women to have a regular point of contact for healthcare [45, 46]. This is especially important in resource-poor settings, where visiting health facilities and interacting with healthcare professionals can increase women’s awareness of related health issues and motivate them to take preventive measures [47]. Studies have also consistently shown that a healthcare provider’s recommendation plays a vital role in determining whether a woman should undergo cervical cancer screening [48].

Women who own mobile phones have increased odds of being screened for cervical cancer compared to their counterparts. This could be because women who own mobile devices demonstrate better health-seeking behaviors, which may include preventive measures such as cervical cancer screening [49].

Women who resided in urban areas had higher odds of cervical screening than those from rural areas. This is in line with the studies conducted in Nepal [50], Ethiopia [30], and Uganda [25]. The possible explanation Urban residents may have important information about cervical cancer screening and access to health care services. Moreover, women from urban dwellers may have better educational backgrounds than their counterparts.

The availability of cervical screening services may be hindered in rural areas compared to urban areas because of the absence of screening centers within walking distance, limited transportation options, and cost of transportation [51, 52]. Additionally, urban areas may offer better opportunities for residents to access health information because of the accessibility of information sources, which could result in differences in screening utilization rates between urban and rural populations.

Women from the Southern Highlands administrative Zones of Tanzania were significantly associated with higher odds of being screened for cervical cancer compared to Eastern Zones; however, women from the Southern and Zanzibar Zones were significantly associated with lower odds of cervical cancer screening. A possible reason for this difference might be that women from the southern highlands of Tanzania are socioeconomically valuable [53], which will help them access education and healthcare services compared with the southern and Zanzibar administrative zones.

This study had both strengths and limitations. This study utilized a weighted pooled nationally representative TDHS survey in Tanzania, and a multilevel binary logistic regression analysis was conducted to obtain reliable estimates and standard errors. The sample size was sufficient to detect the true effects of the independent variables. However, readers should keep in mind the limitations of this study, as it cannot establish a causal relationship between cervical cancer screening and predictors owing to the use of cross-sectional data. Additionally, the outcome variable, cervical cancer screening, was self-reported. This may have introduced the potential for recall bias, which could underestimate the effect size in those who were not able to accurately recall having undergone cervical screening. There is also the potential for reporting bias, resulting in women reporting cervical cancer screening, thereby potentially overestimating the effect sizes.

## Conclusion

Cervical cancer screening among women of reproductive age in Tanzania is low. Our findings identified both individual- and community-level factors associated with cervical cancer screening in women of reproductive age in Tanzania. Women’s age, educational status, employment, household wealth status, visiting health facilities in the last 12 months, owning mobile phones, place of residence, and region were significant predictors of cervical cancer screening uptake. Enhancing awareness campaigns and health education are essential for improving access to cervical cancer screening, particularly in rural areas and among low-income populations. Additionally, integrating cervical cancer screening into existing maternal health services such as contraceptive programs could significantly enhance participation and overall effectiveness. Moreover, to boost cervical cancer screening rates, policymakers and stakeholders must consider the risk factors associated with screening among women, especially in areas where the uptake of cervical cancer screening is low.

## Abbreviations

AOR: Adjusted Odds Ratio
ANC: Antenatal care
DHS: Demographic and Health Survey
TDHS: Tanzania Demographic Health Survey
CI: Confidence Interval; EAs: Enumeration areas
ICC: Intra-cluster Correlation Coefficient
IR: Individual Record
LLR: Log likelihood ratio
LR: = Likelihood ratio
MOR: Median Odds ratio
OR: = Odds ratio
SDG: Sustainable Development Goal
SSA: sub-Saharan Africa
WHO: World Health Organization
